# Direct optical mapping of transcription factor binding sites on field-stretched λ-DNA in nanofluidic devices

**DOI:** 10.1093/nar/gku254

**Published:** 2014-04-21

**Authors:** K. K. Sriram, Jia-Wei Yeh, Yii-Lih Lin, Yi-Ren Chang, Chia-Fu Chou

**Affiliations:** 1 Nano Science and Technology Program, Taiwan International Graduate Program, Institute of Physics, Academia Sinica, 128, Sec. 2, Academia Road, Nankang, Taipei 11529, Taiwan; 2 Department of Engineering and System Science, National Tsing Hua University, ESS New Building, 101, Sec. 2, Kuang-Fu Road, Hsinchu 30013, Taiwan; 3 Institute of Physics, Academia Sinica, 128, Sec. 2, Academia Road, Nankang, Taipei 11529, Taiwan; 4 Research Centre for Applied Sciences, Academia Sinica, 128, Sec. 2, Academia Road, Nankang, Taipei 11529, Taiwan; 5 Genomic Research Centre, Academia Sinica, 128, Sec. 2, Academia Road, Nankang, Taipei 11529, Taiwan; 6 Department of Chemistry, National Taiwan University, 1, Sec. 4, Roosevelt Road, Daan, Taipei 10617, Taiwan

## Abstract

Mapping transcription factor (TF) binding sites along a DNA backbone is crucial in understanding the regulatory circuits that control cellular processes. Here, we deployed a method adopting bioconjugation, nanofluidic confinement and fluorescence single molecule imaging for direct mapping of TF (RNA polymerase) binding sites on field-stretched single DNA molecules. Using this method, we have mapped out five of the TF binding sites of *E. coli* RNA polymerase to bacteriophage λ-DNA, where two promoter sites and three pseudo-promoter sites are identified with the corresponding binding frequency of 45% and 30%, respectively. Our method is quick, robust and capable of resolving protein-binding locations with high accuracy (∼ 300 bp), making our system a complementary platform to the methods currently practiced. It is advantageous in parallel analysis and less prone to false positive results over other single molecule mapping techniques such as optical tweezers, atomic force microscopy and molecular combing, and could potentially be extended to general mapping of protein–DNA interaction sites.

## INTRODUCTION

Transcription factors (TFs) are proteins that bind to specific bases of DNA using DNA-binding domains to carry out the process of transcription (1), which play a major role in the process of transcribing sequential information from DNA to messenger RNA. Thus, mapping TF binding sites is an essential step in understanding the genetic regulatory circuits that control cellular processes. Currently practiced techniques like Chromatin ImmunoPrecipitation with microarray technology (ChIP-on-chip) (2,3) and other recent advancements in ChIP methodology (4,5), such as ChIP-seq, are well established in TF binding site mapping capable of achieving mapping resolution of ∼ 300 nucleotide bases (∼ 100 nm) or better. ChIP-based approach has the advantage for studying *in vivo* DNA–protein interactions in a whole genome perspective. However, when more than one protein is involved in complex formation, ChIP results may not tell if the TF of interest is directly bound to the DNA sequence or through other proteins as a complex. Methods such as electrophoretic mobility shift assay (EMSA) and DNA footprinting are used for identification of TF binding sites mostly *in vitro* (6). Each technique mentioned above has its own advantages and limitations and in most cases, more than one technique is employed to understand DNA–protein interactions (7,8).

In recent years, single molecule approaches (SMA) (9,10) have evolved into powerful ways to study TF binding at the molecular level, which in general are not feasible with the ensemble experiments (11,12). Due to the progress in nanofabrication methods, together with advances in fluorescence single molecule imaging, we are now able to address questions like the mechanism involved in TF binding site identification (13–15) and DNA–protein binding kinetics (16). At present, SMA to study DNA–protein complexes are limited to *in vitro* interactions and a direct comparison with methods like ChIP could be misleading. Nevertheless, SMA can bridge the gap between currently practiced *in vitro* and *in vivo* methods, serving as reliable complementary methods.

Various groups have reported TF binding site mapping using SMA such as optical tweezers (17), atomic force microscopy (AFM) (18) and molecular combing (19). These techniques, nevertheless, suffer from either poor mapping resolution, difficulty with multiplexing or non-specific binding leading to false positive results. Recent studies show promising results for mapping very long genomic DNA molecules (few megabases) using biaxial confined nanochannels (20,21), and effort has been made in using similar structures for DNA–protein complex studies (22,23). Though such highly confined channels are suitable for studies involving negatively charged DNA molecules, they suffer from serious non-specific adsorption when DNA-binding proteins are involved (24). Here, we demonstrated a method that exploits the advantages of bioconjugation, nanofluidic confinement, reversible field-induced DNA stretching and fluorescence single molecule imaging, and analysis to map TF binding sites directly on single DNA molecules (14,22,25). Our approach could overcome the above limitations and drawbacks involved in the existing techniques.

**Figure 1.**
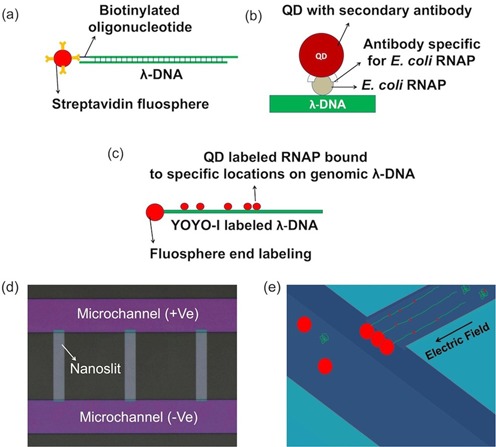
Schematics of the strategy used for direct mapping of transcription factor binding sites: (**a**) streptavidin-functionalized fluosphere conjugated to biotinylated oligonucleotide at the 3′ end of λ-DNA, (**b**) the fluorescent labeling of *E. coli* RNA polymerase (RNAP) through secondary antibody-coated quantum dot (QD) and (**c**) the complete scheme incorporating both (a) and (b), where λ-DNA is post-fluorescently labeled (YOYO-1 dye, green) after being coupled to a fluosphere (large red dot) and complexed with the QD labeled (small red dots) *E. coli* RNAP holoenzyme. (**d**) Optical micrograph of a PSQ-bonded nanofluidic device, which has three parallel 60-nm deep nanoslits (light gray regions) in the middle, connecting the microchannels (magenta regions). (**e**) Schematics of the field-stretched DNA–protein complexes in the nanoslit region.

In this work, we use fluidic devices composed of micro- and nanoregions fabricated in fused silica substrates and are conformably sealed by a polymer-coated coverslip. The nanoregion here is a uniaxial confined nanofluidic slit (nanoslit) with tens of nanometers in depth, which is comparable or less than the persistence length of a double-stranded DNA, i.e. ∼ 50–60 nm (26), to assist a high degree of DNA stretching with nanoconfinement under applied field. TF-bound λ-DNA molecules, coupled with fluospheres (of sizes similar to or larger than the depth of the nanoslits) at one end, were trapped at the micro-nano junction and stretched in the nanoslits in the presence of a small electric field (27–30). Both DNA and proteins were fluorescently labeled to achieve high-resolution mapping of TF binding sites using epi-fluorescence microscopy (Figure 1).

## EXPERIMENT SCHEMATICS

### Device fabrication

Here, we describe the technique used in our work to identify TF binding sites on field-stretched single molecules of fluosphere–DNA–protein complex. Our fluidic device is fabricated on fused silica substrate by a standard two-step photolithography process. First, H-shaped microchannels (100 μm in width and 1 μm in depth) and reservoirs were formed using UV lithography followed by inductively coupled plasma (ICP) etching. A second step UV lithography followed by a short reactive ion etching was carried out to define the shallow nanoslits (200 μm long, 10 μm wide and 60 nm deep) across the H-shaped microchannels.

Through holes were sandblasted on the substrate to form inlet/outlet of loading reservoirs and then the device was conformably sealed with a coverslip using a room temperature polymer (polysilsesquioxane) bonding technique (31,32). Alternatively, the device bonding may be achieved by reverse nanoimprinting (33). Polysilsesquioxane (PSQ) is a Si-based inorganic–organic polymer with a Young's modulus of 800 MPa, thus enabling high-quality bonding for channels of ultralow aspect ratio. Briefly, PSQ is prepared by mixing xylene with Hardsil (Gelest Inc.) in 2:1 ratio, filtered with a 0.45-μm polytetrafluoroethylene (PTFE) membrane filter (Basic Life Inc.) and spun on a piranha (H_2_SO_4_ and H_2_O_2_ in 1:1 ratio) cleaned coverslip (No.1, Goldseal). Then, the polymer is cured at 240°C for 30 min. Both PSQ-coated coverslip and piranha-cleaned chips were exposed to oxygen plasma treatment to enable strong bonding between the chip and the PSQ-coated coverslip through a silanol group condensation process (27). Finally, silica reservoirs were glued to the loading holes using UV curable glue (No. 108, Norland Optical Adhesives). Gold electrodes in contact with the buffer solution filled in each of the four reservoirs form the electrical contacts (see Supplementary Figures S1 and S2 for more details on device fabrication and PSQ bonding).

### Model system

To demonstrate the advantages of our fluidic devices for direct mapping of TF binding sites on long DNA, we used a model biological system, *Escherichia coli* (*E. coli*) RNA polymerase (RNAP) holoenzyme complexed to λ-DNA. *E. coli* RNAP holoenzyme is a 450 kDa protein with five sub-units (34,35). All the five sub-units have been verified by SDS-PAGE experiment (see Supplementary Figure S3) The σ sub-unit (σ70) is responsible for the sequence-specific binding of these proteins to the DNA molecules. The promoter binding sites for RNAP along λ-DNA are well known (36). Previous works using this system have shown the presence of two strong promoters P_R_ and P_L_ and various pseudo-promoters (regions that closely match the promoter sequence) in λ-DNA (17).

### DNA end labeling

The DNA used in the experiments is from λ-phage, a bacterial virus that infects the *E. coli*, with a fully sequenced length of 48,502 bp (37). λ-DNA has complementary, 12-base GC-rich cohesive sticky ends, which enable them to circularize thereby preventing them from being degraded by host endonucleases. We took advantage of these 12-base sticky ends and ligated a complementary 12-base strand with biotin to one of the DNA ends (3′ end in our case). These biotinylated DNA molecules were then coupled to streptavidin-coated fluospheres (Molecular Probes; see Supplementary Information for detailed protocols). These fluospheres at DNA ends, which are slightly larger than the nanoslit depth, help retaining DNA molecules at the micro-nano junctions when external field is applied to stretch DNA. But, this system is not only limited to DNA molecules with sticky ends, e.g. the *cos* sites of λ-DNA. Other DNA molecules with blunt ends (e.g. T7 DNA) can also be modified using a different approach, where biotin tags can be incorporated to the chosen DNA end using terminal deoxynucleotidyl transferase (TdT) or T4 DNA polymerase/Klenow enzyme assisted end labeling (38,39) (see Supplementary Figures S4 and S5 for more details).

Recent publications show the possibility of using uniaxial-confined nanoslits for effective stretching of DNA molecules without any end labeling (21,30). In the present work, end-labeling scheme via fluospheres has been incorporated to stretch tens of DNA molecules in parallel in each nanoslit (Figure 1a); the lack of such scheme makes it impossible. Moreover, it also serves as a positional and orientational reference in mapping the locations of protein molecules bound along the DNA backbone. Earlier work showed that the end -labeling efficiency is dependent on both the length and density of DNA, and for molecules like λ-DNA, it can reach 70% (40). Thus, end labeling proves to be efficient without affecting the device throughput.

### DNA stretching in nanoslits

Once the fluidic channels were filled with buffer containing fluosphere-conjugated DNA, a small DC field (∼2 V/cm) was applied across the microchannels to drive the fluosphere–DNA complexes toward the nanoslit region. Then, a much smaller field (500 mV/cm) was applied so that those DNA molecules with end-labeled fluospheres get trapped at the micro-nano junction. Other non-conjugated DNA molecules normally pass through the nanoslit to reach the other end of the microchannels. The trapped DNA molecules get stretched into the nanoslit region in the presence of the field.

**Figure 2.**
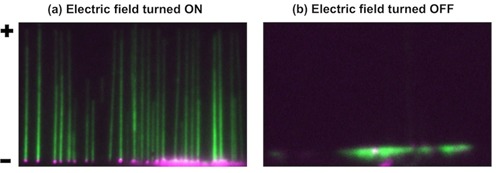
(**a**) Fluorescence microscopy images of λ-DNA molecules (green) end labeled with 40 nm (magenta) streptavidin transfluospheres (excitation/emission at 488/645 nm, Invitrogen Molecular Probes), stretched in the nanoslit (60 nm deep) with an applied electric field. (**b**) DNA molecules recoiled back into the microchannels when the electric field is turned off (see Supplementary Movie M2).

There are three parallel nanoslits in our device (Figure 1d), which are arranged in such a way that only one of the nanoslits is in the field of view during observation. Each slit is 10-μm wide and, in average, has 20–30 DNA molecules fairly separated from each other during each observation. All DNA molecules are arranged in parallel with a common reference point (micro-nano junction) and the stretching and relaxation of fluosphere–DNA complexes may be simply achieved by switching the field on and off, respectively (Figure 2 and Supplementary Movie M1).

## MATERIALS AND METHODS

### Sample preparation

**Figure 3.**
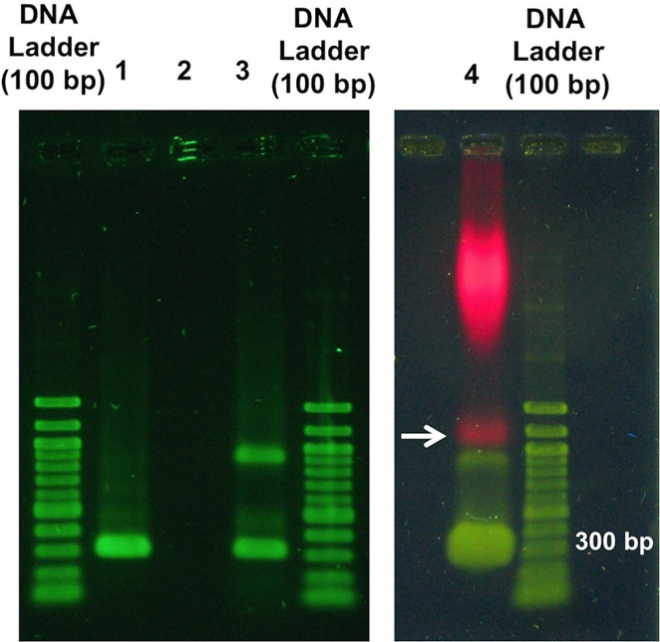
Gel shift assay to confirm the formation of DNA–RNAP holoenzyme complexes. Lane 1: DNA alone (310 bp PCR product with P_R_ promoter region); Lane 2: *E. coli* RNAP holoenzyme alone; Lane 3: DNA + *E. coli* RNAP holoenzyme complex. Results in Lane 4 (indicated by an arrow) of the gel shows super-shift assay results for DNA–RNAP holoenzyme complex labeled with a quantum dot through primary antibody–secondary antibody complex scheme.

In our experiments, RNAP molecules were labeled with quantum dots (QDs) through a primary antibody (AB)–secondary antibody coupling scheme. A primary antibody (Mouse monoclonal, WP001, Neoclone) that binds specifically to one of the sub-units of RNAP was chosen. Then, a QD (655 nm Anti-mouse IgG, Invitrogen) with a secondary antibody against the chosen primary antibody was used. The AB–QD complexes were prepared by mixing AB and QD in 1:1 ratio. Meanwhile, DNA–RNAP complexes were prepared separately using formaldehyde crosslinking mechanism (41). After this step, DNA–RNAP complex solution was mixed with AB–QD complex solution to label the RNAP molecules. Complex formation for chosen buffer conditions was verified by gel shift assay experiments, which were conducted using 1% Agarose gel and a 310 bp PCR fragment with P_R_ promoter region used to test the complex formation. Two controls were prepared with no RNAP molecules in one (Lane 1) and no DNA molecules in the other (Lane 2). An assay was also performed before (Lane 3) and after (Lane 4) labeling DNA–RNAP complexes with QDs through primary antibody–secondary antibody coupling scheme (Figure 3, see Supplementary Information for more details).

For single molecule experiments, the whole complex was diluted in observation buffer (0.5× TBE, 10% (w/v) glucose, 2.5% (w/v) PVP and 0.1% (v/v) Tween 20) containing an oxygen scavenging system (50 μg/ml glucose oxidase, 10 μg/ml catalase and 0.5% (v/v) β-mercaptoethanol or BME) and DNA molecules were labeled with YOYO-I nucleic acid stain (1 dye/5 bp) for easy observation prior to loading sample in the reservoirs of the fluidic devices (42). Though in real applications, DNA labeling is not necessary, but as a demonstration, it will be more convincing to see both DNA and proteins and thus we labeled DNA molecules using an intercalating dye, a common practice in this field. Earlier studies indeed suggest that intercalating dyes like YOYO-I might inhibit enzyme activities (43). To avoid such problems in our experiments, YOYO-I nucleic acid stain was added *after* the formation of DNA–RNAP complexes and immediately before observation, thus not expected to have any effects on DNA–RNAP complex formation efficiency, as demonstrated by EMSA using similar dyes for post staining of gels to observe other DNA–protein complexes (44). It is noted that for practical applications, DNA labeling using intercalating dyes could be replaced by double end labeling of DNA or by sequence-specific binding proteins for length or position reference (45–47).

Glucose, glucose oxidase and catalase together form the oxygen scavenger system and BME is also an anti-photobleaching agent, thus increasing the observation time. PVP helps reduce electro-osmosis and passivate the channel surface to minimize non-specific binding of proteins and DNA molecules (30,48) (Supplementary Movies M3 and M4). The DNA concentration in the final solution is ∼ 0.1 ng/μl (see Supplementary Information for more details on sample preparation and surface passivation).

### Driving DNA–protein complexes into nanoslits

Again, once the fluidic channels were filled with buffer containing fluosphere–DNA (now fluorescently labeled)–protein (RNAP–AB–QD) complexes, a small DC field (∼2 V/cm) was applied across the microchannels to drive the fluosphere–DNA–protein complexes toward the nanoslit region. Then, a smaller field (500 mV/cm) was applied so that those DNA molecules with end-labeled fluospheres get trapped at the micro-nano junction. All fluosphere–DNA–protein molecules are arranged in parallel with a common reference point at the micro-nano junction. Thus our system facilitates multiplexing, which may not be easily accessible with other reported systems such as optical tweezers, AFM, etc.

**Figure 4.**
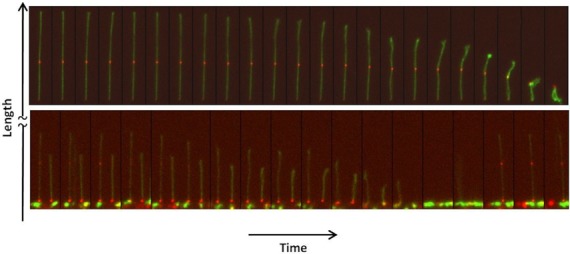
Time-lapse images showing the stretching and recoiling of λ-DNA with bound QD-labeled *E. coli* RNAP complex by applied electric field in the nanoslit (see Supplementary Movies M2–M4).

One important requirement for uniform DNA stretching is that no loops are formed. Formation of loops due to thermal fluctuations can be avoided if the channel dimensions are close to or less than the persistence length of the DNA (*L*_p_ ∼ 50–60 nm). In such cases, the loop formation under our nanoslit confinement is minimized as the bending energy *U* = *πk*_B_*T*(*L*_p_/2*R*) required to form a loop is greater than that of the thermal energy *k*_B_*T*, where *k*_B_ is the Boltzmann constant, *T* is the absolute temperature and *R* is the radius of curvature of the loop or bend (in our case the nanoslit height is 2*R* for an 180° bend) (49,50). One other factor that plays a role is the strength of the applied electric field. When the field strength is low, the weakly stretched DNA molecules show increased Brownian fluctuations. But at higher field strengths, the molecule becomes more stretched and the inhomogeneity in tension along the DNA chain is reduced. Top panel of Figure 4 shows DNA stretching–relaxation over time, and one can see that the extension is more uniform (far left image) for maximum applied electric field when compared to smaller field (moving from left to right). There is indeed a threshold value of applied electric field, above which the changes in stretching are very minimal (51).

Also, our nanoslits are 200 μm long and can be extended freely to enable experiments involving very long genomic DNA molecules. Further, the false positive results of RNAP binding sites may be excluded simply by switching the field reversibly (Figure 4 and Supplementary Movies M5–M6). The positive RNAP binding sites will remain during the stretching and relaxation operation. This feature overcomes some drawbacks of the earlier single molecule methods for studying DNA–RNAP complexes, such as molecular combing or AFM studies, in which the real DNA–protein complex cannot be easily distinguished from the false ones (52).

### Optical setup

Fluorescence imaging was performed on a Leica DMI 4000-B microscope with a mercury lamp and filters (470/40 nm band-pass/585 nm dichroic/ 655/40 nm long-pass filter), via a 100× oil lens (Plan-Apo, N.A. 1.4), an additional magnifier (1.6×) and an EMCCD (Ixon 897, Andor). A split view system (488 nm band-pass/585 nm dichroic/655 nm long-pass filter, Optical Insights) was placed in front of the EMCCD to split the signal from DNA (green) and end-labeled fluospheres and QDs (red). Most of the previously reported works related to single molecule DNA–protein complex studies have used total internal reflection fluorescence (TIRF) system in their experiments to achieve a better signal-to-noise ratio (53). Here, we have shown that our fluidic device with sub-100 nm depth helps to achieve better signal-to-noise ratio with a regular epi-fluorescence microscope, as all signals from the nanoslits are within the depth of focus.

## RESULTS AND DISCUSSION

### Particle localization and histogram plot

**Figure 5.**
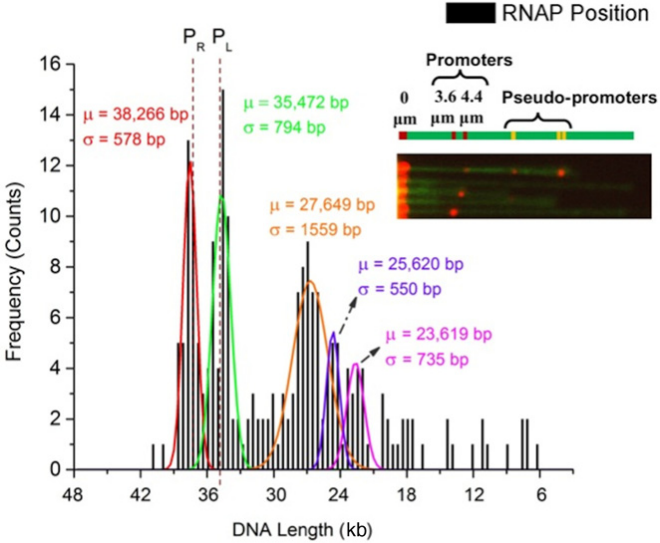
Localization of RNAP molecules bound to λ-DNA stretched in the nanoslit. Histogram shows the values obtained from our experiments (∼200 molecules) for known promoters (at 38 266 and 35 472 bp loci) and pseudo-promoter (at 27 649, 25 620 and 23 619 bp loci) regions of *E. coli* RNAP holoenzyme. The dotted lines represent actual promoter regions P_R_ (38 003 bp) and P_L_ (35 602 bp) of λ-DNA, obtained from (17,36). Our experiments were carried out with the 3′ end-labeled (streptavidin fluosphere) as the reference point. Inset shows assorted images of DNA–RNAP complexes with all five binding sites.

After the experimental conditions were optimized in terms of fluidic device fabrication and fluosphere–DNA–RNAP complex formation, we repeated the experiments to get statistical data from our experiments. Images were collected and analyzed and a histogram is plotted with results from ∼ 200 fluosphere–DNA–RNAP complexes (Figure 5). DNA contour length increases from 16.5 μm to around 22 μm due to YOYO-1 labeling (28), and 87% of DNA stretching was observed in our experiments (29,30,49). High-precision localization of the QDs was carried out using centroid localization method (54). This was carried out by deconvolving the collected distribution of photons to the point-spread function (PSF) of the system. With this method, we could achieve localization precision around 2.5 nm for a typical QD (see Supplementary Figure S6 for more details).

Phage λ-DNA has two promoter regions for *E. coli* RNAP holoenzyme. The P_R_ and P_L_ promoter regions are located near the 38 000th and 35 600th bases, respectively, which correspond to 3.55 μm and 4.40 μm from the fluosphere-labeled 3′ end. Our histogram plot shows that our results for these two promoter regions are in accordance with the expected RNAP binding sites along phage λ-DNA backbone. In addition to these two peaks, we also see three more significant peaks around 7–9 μm (23 000 to 28 000 bases) regions. DNA molecules that have the end-labeled fluosphere and at least one RNAP–QD bound to stretched DNA were considered for construction of position histograms. Only those DNA molecules of length 9 μm or longer were taken into account to obtain the binding frequency to avoid discrepancy in obtained histogram.

Earlier studies from various groups have shown that *E. coli* RNAP holoenzyme indeed has the affinity to form complexes in specific regions other than the promoter sites, the so-called pseudo-promoters for these promoter-like regions (17,55). Our results also show that binding events are more frequent (∼ 45%) for the two strong promoter regions when compared to the three pseudo-promoters (∼ 30%) and, generally, the distributions are slightly broader for the three pseudo-promoter regions. Previous study shows that these pseudo-promoters generally occur at interior sites rather than at the extremities (55). Literature studies also suggest that unlike non-specific interactions, complexes formed at pseudo-promoter sites exhibit strong binding and that they are able to initiate transcription, but can be distinguished on the basis of their rate of formation (55). Specific reasons behind the existence of pseudo-promoters are not known. But, earlier works suggest that these regions may have a role in the kinetic mechanism leading to promoter binding (55). Specific conditions that influence the formation of complexes in these promoter-like regions are beyond the scope of this study.

A multi-peak Gaussian fit of the obtained histogram shows that the strong promoter binding sites P_R_ and P_L_ can be determined with error (difference between actual promoter site and mean value obtained from our experiments) less than 300 bp (100 nm) and standard deviation (obtained from fitted Gaussians) within 800 bp (250 nm). This can be improved when more molecules are used for mapping. Presence of the end-labeled fluospheres is advantageous from the point of view of mapping binding site locations as it serves as a reference to identify the orientation of DNA as well as in improving the mapping resolution.

### Comparison with earlier studies

Here, we further elaborate the issues involved in other techniques for the mapping of DNA–protein binding sites. In general, AFM has the advantage of directly observing single molecules without the need for any fluorescent labels, but lacks multiplexing ability and needs multiple scanning over selected regions of the molecule to read fine details (18). Dual optical tweezers combined with fluorescence imaging can be used for TF binding site mapping, but lack the ability to quickly read many molecules, thereby making it difficult to obtain statistical data (17).

Molecular combing of DNA–protein complexes is another option where many DNA–protein complexes can be stretched along a hydrophobic substrate (19,56). This method is simple, but studies have shown that the force exerted on the DNA molecules results in over-stretching, altering the base stacking structure of the DNA, hindering normal DNA–protein interactions (52). Also, the hydrophobic nature of the surface leads to increased non-specific binding of proteins.

Alternatively, flow-induced stretching methods may overcome the disadvantages involved in molecular combing, where DNA–protein complexes were stretched on a positively charged poly-l-lysine surface by electrostatic interaction (57). In this way, the binding locations of T7 RNAP were mapped along stretched phage T7 DNA molecules. Some drawbacks such as inability to identify DNA orientation and enzymatically incorporating reference tags using DNA methyltransferase may improve non-uniformity in DNA extension factor (47). However, non-specific adsorption of QDs to the poly-l-lysine surface could still be a potential issue, as this method has to rely only on their measurements to distinguish real complexes from false positive results. Such false positive results may pose problems when unknown DNA–protein complexes were mapped.

First effort to stretch many DNA molecules in parallel was achieved using microfabricated gold patch using gold–biotin–streptavidin–biotin–DNA conjugation or gold–thiol–DNA linkage (58). The drawback with this method is that proteins and other fluorescent particles also adsorb to the gold patch. Modified versions of these devices, called “nanofabricated racks”, may also be used to anchor one or both ends of DNA to the microfluidic sample chamber (59,60). Though not being demonstrated, potentially it could be used in a multiplex fashion to map TF binding sites along stretched genomic DNA molecules. However, one drawback of the double-tethered DNA curtain design is the selective but non-specific adsorption of end-modified DNA molecules to the relatively large exposed surface of metallic pentagons formed using electron beam lithography. As no reference tags (either on DNA ends or along the DNA backbone) have been used for protein binding studies, it may also result in reduced mapping accuracy. In their system, DNA–protein interactions take place on chip, with fluorescently labeled DNA molecules already stretched using flow (61). Although this helps to perform protein binding-kinetics studies, DNA is not in its physiologically resembled coiled state. Earlier work by Harada *et al.*, using dual optical tweezers has shown that the interaction of proteins with DNA molecules in stretched state and relaxed state is different (17). In our work, DNA–protein complex formation takes place in an eppendorf tube, though it is not *in vivo* but in its physiologically resembled coiled state.

Mapping megabase long DNA molecules using nanochannel devices has been demonstrated by various groups (20,21,46). One such work involves 45×45 nm^2^ nanochannels to map 4.7-Mb long, nick-labeled bacterial artificial chromosomes (20). Their method proves to be high throughput, when compared to earlier DNA mapping techniques. However, DNA–protein complex were tried using similar devices but without much success (22). This is because while negatively charged DNA repelled from channel walls like SiO_2_, proteins tend to adsorb non-specifically to the channels. As effective stretching can only be achieved in a nanochannel with width and depth less than the persistent length of DNA (50 nm) (33), in such cases, all four walls of the nanochannels are in close interaction with DNA–protein complexes (15–20 nm) and the use of QDs or fluospheres (∼20 nm) to label proteins will exacerbate the problem. Some proof-of-principle works have showed passivation techniques to minimize non-specific adsorption of proteins and QDs/fluospheres to channel walls, but radically increase the complexity of such experiments (24). On the other hand, uniaxial confined nanoslits involve only two surfaces in proximity to DNA–protein complexes and our experiments show very minimal non-specific adsorption of proteins and QDs/fluospheres for extended periods of time (see Supplementary Movies M5 and M6).

Currently practiced genome sequence or mapping methods require the whole genomic DNA molecules (tens of kilobases to megabase long) to be divided into comparatively small fragments (order of kb). Rest of the work relies on bioinformatics methods to assemble the obtained data to get whole genome information. To our knowledge, there is no work available on single molecule DNA–protein interactions that demonstrate the use of very long genomic DNA molecules. But, some work on DNA mapping has been done recently, for example by Zhou *et* *al*. on restriction digestion mapping (62) and Lam *et al.* on nicking site mapping (20). So, a practical approach to use our platform to map longer genomic DNA molecules could be an extension of DNA mapping methods referred above (20,62), obtaining overlapping field-of-view (FOV) images to cover the whole genomic DNA length, and stitch the images together to get complete information of protein binding sites. Such an approach will also have its drawbacks (data redundancy, stitching error, etc.). In this case, in addition to the end-labeled fluosphere as reference, more reference sites can be incorporated in the DNA backbone using nick labeling (20,46) or methyltransferase assisted labeling (47). Weiss *et al.* have used multiple reference approach to improve protein binding site mapping resolution, but the same technique could potentially be used to map protein sites along very long genomic DNA molecules (47).

## CONCLUSIONS

In summary, we have demonstrated a simple and robust nanofluidic platform that can be used for effective identification of protein binding sites along field-stretched single DNA molecules. Shown here in this study is the *E. coli* RNAP TF mapping on phage λ-DNA where two promoter and three pseudo-promoter binding sites are identified, consistent with the literature findings. Our results show that our device is suitable for multiplexing and with accuracy comparable to common techniques like ChIP-on-chip for TF binding sites mapping, without the need of using sophisticated optical setup such as TIRF microscopy, making our platform potentially a complementary technique to conventional ChIP-on-chip methods. The fluosphere end-labeling scheme at 3′-DNA end provides a positional and orientational reference in mapping the locations of protein molecules bound along the DNA backbone, without compromising on the throughput. Furthermore, reversible operation of DNA stretching/recoiling using electric field helps distinguish real DNA–protein complexes from false positive events, which is generally not accessible with other methods such as AFM, molecular combing, etc. Room temperature bonding using PSQ opens up the possibility to reuse our devices, thus making it advantageous over other commonly used bonding methods.

We also envision that our device may be applicable to study the mapping of DNA nicking sites (20,46), RecA-promoted homologous pairing and strand exchange (63), cisplatin-induced DNA condensation (64), etc., along stretched single DNA molecules, and to verify the predicted, yet unobserved, TF binding sites in yeast genome and mammalian cells (65–67). Though our current demonstration uses *in vitro* DNA–protein complexes, our device may open up the possibility for a lab-on-chip device in which *in vivo* complexed DNA–protein samples can be extracted from a cell and protein binding sites mapped (68,69). Also, this system is not only limited to single protein systems but could potentially be used for complex systems where more than one protein is involved (70–72). It is noted that complexities are involved in extending a demonstration using model λ-DNA system to a real genomic DNA system like *E. coli*, yeast, etc. Shearing of DNA into smaller fragments during multiple sample preparation steps, increased complexity due to involvement of multiple proteins in higher order systems, challenges and drawbacks involved in imaging and analysis of megabases of DNA lengths, etc. has to be sorted out. We conclude that we have developed a platform that is versatile and may be used for simple and quick analysis of long single DNA-protein complexes.

## SUPPLEMENTARY DATA

Supplementary Data are available at NAR Online, including [73–79].

SUPPLEMENTARY DATA
