# Supplementary material for: Direct optical mapping of transcription factor binding sites on field-stretched λ-DNA in nanofluidic devices
Source: Nucleic Acids Res. 2014 Apr 21;42(10):e85. doi: 10.1093/nar/gku254 (PMC4041428; doi:10.1093/nar/gku254)
Supplement: SUPPLEMENTARY DATA [file supp_42_10_e85__index.html]

Direct optical mapping of transcription factor binding sites on field-stretched λ-DNA in nanofluidic devices — SUPPLEMENTARY DATA 

# Direct optical mapping of transcription factor binding sites on field-stretched λ-DNA in nanofluidic devices

## SUPPLEMENTARY DATA

**Files in this Data Supplement:**

- SUPPLEMENTARY DATA
- SUPPLEMENTARY DATA
- SUPPLEMENTARY DATA
- SUPPLEMENTARY DATA
- SUPPLEMENTARY DATA
- SUPPLEMENTARY DATA
- SUPPLEMENTARY DATA
