# Supplementary material for: Direct optical mapping of transcription factor binding sites on field-stretched λ-DNA in nanofluidic devices
Source: Nucleic Acids Res. 2014 Apr 21;42(10):e85. doi: 10.1093/nar/gku254 (PMC4041428; doi:10.1093/nar/gku254)
Supplement: SUPPLEMENTARY DATA [file supp_gku254_nar-02732-met-k-2013-File007.pdf]

Supporting Information

Direct Optical Mapping of Transcription Factor Binding Sites on Field-Stretched Lambda DNA in Nanofluidic Devices

K. K. Sriram [a,b,c], Jia-Wei Yeh [c, $], Yii-Lih Lin [a,c,f], Yi-Ren Chang [c], Chia-Fu Chou [c,d,e,*]

[a] Taiwan International Graduate Program, Institute of Physics, Academia Sinica, 128, Sec. 2, Academia Road, Nankang, Taipei 11529, Taiwan.
[b] Engineering and System Science Department, National Tsing Hua University, ESS New Building, 101, Sec. 2, Kuang-Fu Road, Hsinchu 30013, Taiwan.
[c] Institute of Physics, Academia Sinica, 128, Sec. 2, Academia Road, Nankang, Taipei 11529, Taiwan.
[d] Research Centre for Applied Sciences, Academia Sinica, 128, Sec. 2, Academia Road, Nankang, Taipei 11529, Taiwan.
[e] Genomic Research Centre, Academia Sinica, 128, Sec. 2, Academia Road, Nankang, Taipei 11529, Taiwan.
[f] Department of Chemistry, National Taiwan University, 1, Sec. 4, Roosevelt Road, Daan, Taipei 10617, Taiwan.

*** Corresponding Author:** Dr. Chia-Fu Chou. Tel: +886-2-2789-6761 Fax: +886-2-26510704; Email: cfchou@phys.sinica.edu.tw

[$] Present Address: School of Applied and Engineering Physics, Cornell University, Ithaca, NY, 14850, U.S.A.

Materials and Methods:

Device Fabrication:

The device fabrication process flow is summarized in Fig. S1. The micro-nano fluidic channels were fabricated on fused silica wafers (Semiconductor Wafers Inc.) using standard photolithography and dry etching techniques. Photolithography is carried out in two steps to obtain the microchannel features and nanoslit depth. Microchannel features defined by first step UV exposure (S1813 positive photoresist spun at 4000 rpm for 25 seconds, baked at 110°C for 90 seconds, UV exposed at 10 W/cm^2 power for 8.5 seconds and developed using MF319

photoresist developer for 15 seconds) were etched using Inductively Coupled Plasma (ICP, RIE-10ip, Samco) machine. A mixture of $CHF_3/CF_4/O_2/Ar$ gases at flow rates 50/33.3/6.7/30 sccm and bias/RF power 700/300W were used for 2 min and 30 seconds. Surface profiler (Alpha step IQ, KLA Tencor) measurements confirmed features of depth around 1μm.

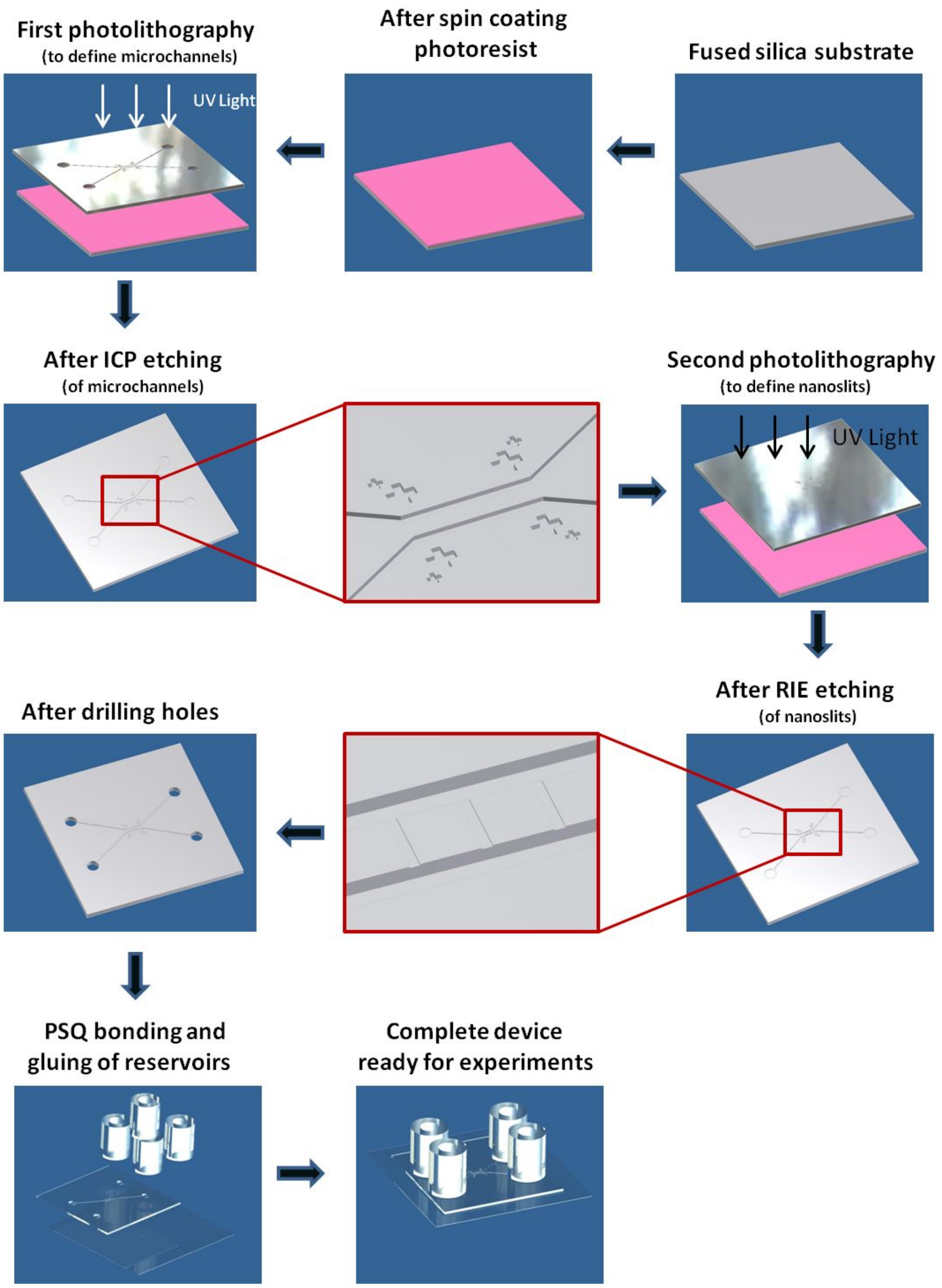

Figure S1. Fabrication process flow of nanoslit devices used in our experiments.

Nanoslit features defined by second step UV exposure were etched using a Reactive Ion Etching (RIE Plasmalab 80+, Oxford Instruments) machine. An initial de-scum process using 100 sccm O_2, 300 mTorr pressure and 150W RF power for 3 minutes to remove any residual photoresist layer in the nanoslit regions is followed by a brief etching using CHF_3/O_2 mixture at flow rates 85/6 sccm, pressure 100 mTorr and RF power of 70W for 8 minutes. Surface profiler and atomic force microscopy (Nanoscope III, Veeco) measurements showed the nanoslit depths are around 60 nm (Fig. S2a). To be noted, as the slit width (10 μm) and length (200 μm) are big enough to be defined by a simple photomask through direct UV exposure of the photoresist and developing, the nanoslit height (or depth) was merely defined by the RIE etching process without complicated and expensive nanolithography process, such as electron-beam lithography. Alternatively, a wet-etching process would work due to the ultralow aspect ratio ($6x10^{-3}$) of our nanoslit.

Above-mentioned fabrication steps were carried out on a 4" fused silica wafer to facilitate batch processing. Each fluidic device is 14x14 mm^2 and thus a 4" wafer can yield nearly 25 working chips. The 4" wafer was subjected to dicing to obtain individual fluidic devices. Then, loading holes were drilled using a sand blaster and the devices are ready for bonding.

(a)

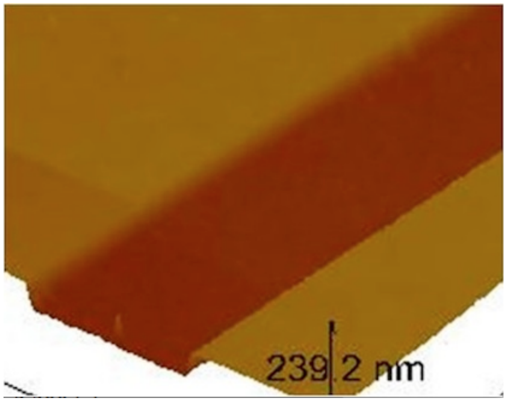

(b)

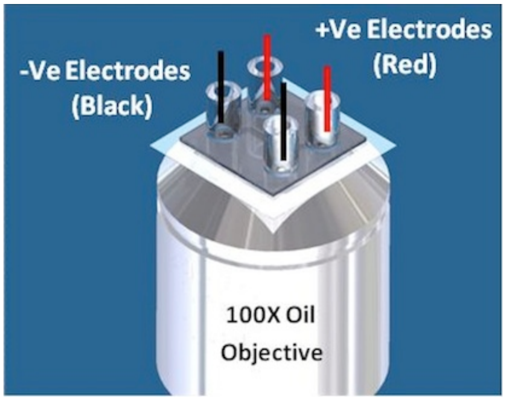

Figure S2. (a) AFM image of the 200 μm long, 10 μm wide and 60 nm deep nanoslit region. (b) Schematic representation of the experimental layout. Four gold electrodes are inserted into the reservoirs, which are filled with buffer containing fluorescently labeled DNA-protein complexes. A small DC voltage is applied to drive the DNA-protein complexes from the reservoirs to the nanoslit region, where they are observed using an inverted epi-fluorescence microscope.

PSQ Bonding Details:

Each diced chip is then bonded using a glass coverslide coated with polysilsesquioxane (PSQ) polymer layer (1). Fabricated chips were thoroughly cleaned using acetone/IPA/de-ionized water and dried. Glass coverslides (No.1, gold seal, 25x25 mm^2) and chips were then subjected to piranha cleaning (H_2SO_4 and H_2O_2 in 1:1 ratio) for 15 minutes to render the surface hydrophilic. PSQ solution was freshly prepared before experiments by mixing Xylene and Hardsil (AP grade, Gelest Inc.) in 2:1 ratio. This mixture was then filtered using a 0.45 μm PTFE membrane (Basic Life Inc.). Piranha cleaned coverslides were coated on one side with PSQ (3000 rpm for 30 seconds) and baked at 240°C for 30 minutes. Then, the PSQ coated surface and the fluidic chips were subjected to O_2 plasma (17 sccm O_2, 50W RF power, 0.18 mbar pressure) for 1 minute. The PSQ coated surface was placed on top of the fluidic device and a small pressure was applied using tweezers to facilitate smooth bonding. Finally, loading reservoirs were glued to the substrate using UV curable glue (No.108, Norland optical adhesives). With this, the device is complete and ready for experiments (See the layout in Fig. S2b).

PSQ bonding is advantageous over other commonly used bonding methods like fusion bonding, anodic bonding etc. as it can be carried out at room temperature, making it suitable for wide variety of applications. One other advantage is that it supports de-bonding and re-bonding, thus making it possible to reuse the devices. For this, bonded devices were soaked in Dynasolve 210 (Dynaloy Inc.) with ultra-sonication for 2-3 hours or overnight without sonication. After this process, PSQ coated coverslides separate easily from the fused silica chips and further cleaned with IPA, rinsed thoroughly with DI water and dried using a nitrogen gun. Now, the chips are ready to be reused and PSQ bonding procedures mentioned above can be carried out. The same chip can be reused effectively for 2-3 times without compromising the bonding quality.

SDS-PAGE and Western Blot Experiments:

SDS-PAGE experiments were done to check the quality of the RNAP samples before experiments. Later, Western Blot experiments were conducted to see if chosen primary antibody can complex with the RNAP.

SDS-PAGE Experiments:

We used a 6% polyacrylamide gel for this experiment. 5.3 ml dH_2O, 2 ml of 30% acryl-bis acrylamide mix, 2.5 ml of 1.5M Tris pH 8.8, 0.1 ml of 10% SDS, 0.1 ml of 10% ammonium persulfate and 0.008 ml TEMED were mixed in the above mentioned order. This solution was poured into a rack (0.75 mm thick, BioRAD) to 3/4 of the rack. Alcohol was sprayed on top to avoid evaporation of solution and the gel was allowed to set for around 30 minutes at room temperature. This layer forms the running gel. Any remaining alcohol was drained and a mixture of 3.4 ml H_2O, 0.83 ml of 30% acryl-bis acrylamide mix, 0.63 ml of 1.5 M Tris pH 6.8, 0.05 ml of 10% SDS, 0.05 ml of 10% ammonium persulfate and 0.005 ml TEMED was poured to cover the remaining 1/4 of the rack. A plastic comb was placed to define the lanes. Again, the gel was allowed to set for 30 minutes at room temperature. This second layer forms the stacking gel.

SDS-PAGE experiments were run using 1X running buffer (Tris/Glycine/SDS buffer 10X stock, #161-0732, BioRAD). Sample was prepared by mixing 1μl *E. coli* RNA polymerase holoenzyme (0.5 μg) with 2.5 μl of 6X sample buffer and 6.5 μl of Tris buffer, 20 mM, pH 8.0 to obtain 10 μl total volume. This sample was mixed well and heated at 100^oC for 10 minutes using a heating block followed by a quick spin to denature the proteins. The gel was placed in the column and filled with 1X running buffer. 10 μl of the RNAP sample prepared was loaded into one of the lanes and one lane on either side was filled with a protein marker. Electrodes were connected and the gel was allowed to run at 200 V, 400 mA for 45 minutes. The gel was then allowed to soak in coomassie blue stain for 30 minutes and the results were obtained.

Our results showed two distinct bands around 175 KDa which matches with the size of β and β' sub-units of *E. coli* RNA polymerase holoenzyme. Clear band around 80 KDa was confirmed to be the σ sub-unit and we also could see a band for α sub-unit. These results also match with the datasheet provided by the supplier (Epicentre Biotechnologies). Presence of σ sub-unit confirms that the product used was a holoenzyme (Core enzymes of *E. coli* RNA polymerase lack σ sub-unit).

<u>Western Blot Experiments:</u>

RNAP sample was prepared and SDS-PAGE experiment was done using the protocol described above. After this step, the gel was soaked in 1X transfer buffer (Tris/Glycine buffer, #161-0734, BioRAD) for 1 hour at room temperature. In a separate container, filter papers and nitrocellulose (NC) membrane for Western Blot experiments were also soaked in 1X transfer

buffer for 1 hour at room temperature. Membrane transfer process was carried out at 24 V for 1 hour to transfer the information from the gel to the NC membrane. Then, membrane was washed with dH2O and then soaked in blocking buffer (1X PBS, 0.1% v/v Tween-20, 5% w/v non-fat milk powder) at room temperature for 1 hour, with constant shaking. Then, NC membrane was washed in 1X PBST buffer (1X PBS, 0.1% v/v Tween-20).

10 µl of primary antibody (Mouse monoclonal, WP001 clone, specific to β' sub-unit, Neoclone Biotech) was added to 10 ml blocking buffer (1000 times diluted) and NC membrane was soaked in this solution and allowed to sit at 4^oC overnight with constant shaking. Then, NC membrane was washed in 1X PBST buffer to remove any unbound primary antibody. 4 µl of 1 µM QDs (Qdot 655 goat $F(ab')_2$ anti-mouse IgG conjugate H+L, Q11022MP, Invitrogen) were added to blocking buffer and NC membrane was soaked for 1 hour at room temperature, with constant shaking. Then, the membrane was taken out and washed again in 1X PBST buffer to remove any unbound QDs. Later, results were observed using a UV-transilluminator. A sharp red band observed around 175 KDa showed that the primary antibody was capable of binding to the β' sub-unit of the RNAP as expected.

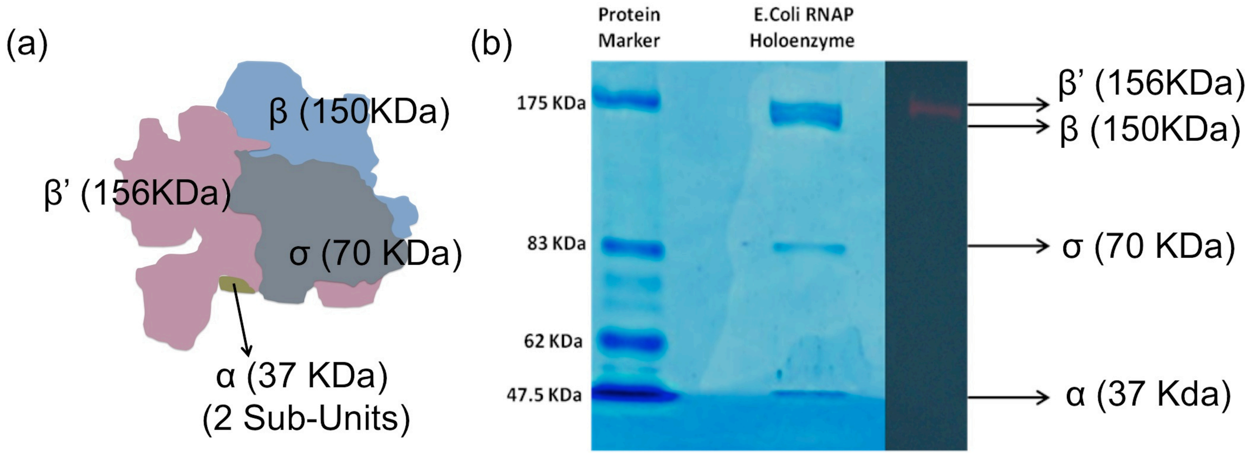

Figure S3. (a) Shows *E. coli* RNA polymerase holoenzyme sub-units (Figure adopted and modified from Finn, R.D *et al.*) (2). (b) SDS-PAGE experiment shows all the sub-units present in the *E. coli* RNA polymerase holoenzyme. The right most column shows the β' sub-unit of the RNA polymerase labeled with quantum dots (655 nm Anti-mouse IgG quantum dots, Invitrogen) using primary antibody (Mouse monoclonal antibody, WP001, Neoclone) as the linker. This experiment was conducted by transferring the Western blot results to a nitrocellulose membrane followed by the QD labeling reaction.

Gel-Shift Assay:

Gel shift assay was done to optimize the conditions for DNA-RNAP complex formation. A shift assay including the primary antibody and secondary antibody conjugated QD was also done to get some insights on DNA-RNAP-AB-QD complex formation conditions.

A 310 base-pair PCR fragment with P_R promoter region (37974 – 38032 bases from the 5' end) was used for these experiments. 1% Agarose gel in 1X TBE buffer was used in all experiments. 1 μl of 310 base-pair PCR product (110 ng/μl) was mixed with 0.5 μl of RNAP (0.5 μg/μl) in 2 μl of 5X CLB buffer (0.25 M HEPES-NaOH pH 8.0, 0.5 M NaCl, 25 mM $MgCl_2$, 25% glycerol) in a total volume of 10 μl. Two controls were prepared with no DNA molecules in one and no RNAP molecules in the other. First, all the solutions were incubated at 37°C for 15 minutes. Then, 1 μl of 37% formaldehyde (F8775, Sigma Aldrich) was added to the above solution, mixed gently and incubated at 4°C for 30 minutes. Formaldehyde reaction was quenched by adding equal amounts of tris buffer. DNA markers were loaded on the lanes at the extremities and sample 1 (DNA alone) was loaded in lane 1, sample 2 (RNAP alone) was loaded in lane 2 and sample 3 (DNA-RNAP complex) was loaded in lane 3. The lane loaded with DNA-RNAP mixture showed a shifted band, which confirmed the formation of complexes.

A similar assay was done with 0.5 μl of AB-QD complex (0.5 μM) added to the DNA-RNAP complex and incubating it at room temperature in a dark place for 45 minutes. Results showed a super-shifted band for DNA-RNAP complex alone labeled with quantum dots. There were some non-specific complexes too, but the results from the specific complexes were distinct from the non-specific ones (See Fig. 3 in main article).

Sample Preparation

Fluosphere end-labeling of λ-DNA:

This process involves multiple steps like ligation of biotinylated primers to DNA ends, removal of unbound oligonucleotides and binding streptavidin fluospheres to biotinylated DNA molecules. We followed a method similar to that of Perkins *et al.* (3) All the steps are discussed in detail here.

Ligation of biotinylated primers to λ-DNA:

λ-DNA has complementary, 12 base pair overhanging sections at each end. This is used to our advantage, to label one end of the DNA molecule with a fluosphere. This fluosphere labeling can help us trap the DNA molecules in the micro-nano interface, thus stretching the DNA molecule in the nanoslit region, in presence of an applied electric field. Moreover, it facilitates DNA orientation identification and RNA polymerase mapping with improved resolution.

λ-DNA used in our experiments was purchased from NEB. The reported stock concentration of the DNA is 500 ng/μl. 12 base pair biotinylated oligonucleotides, complementary to the 3' end of the λ-DNA were purchased from MDBio Inc., Taiwan. An aluminum heating block is pre-heated to 65^oC. 50 μl of λ-DNA (500 ng/μl, NEB) was mixed with 40 μl of biotinylated oligonucleotides (100 μM, MDBio Inc.) in presence of ligase buffer (10X concentrations, NEB) in 400 μl reaction volume.

Above mentioned solutions were mixed gently using a pipette with wide opened tips to avoid DNA fragmentation. Then, the mixture is incubated at 65^oC for 5 minutes. Later, it was allowed to sit at room temperature for few hours to allow addition of oligonucleotides to the DNA ends. DNA ligation is carried out by adding 2 μl of DNA ligase (NEB) and 4 μl of 0.1 M ATP to this solution and incubating it at 16^oC overnight. After the ligation step, the solution is heated again at 65^oC for 10 minutes to inactivate the ligase and hopefully unhybridize any non-ligated oligonucleotides, which were removed in the following step.

Removing Unbound Oligonucleotides:

The unbound oligonucleotides from the previous step were removed, as it would interfere with the fluosphere labeling step in the next step. The reason is that these oligonucleotides are much smaller compared to the ligated λ-DNA molecules and can diffuse faster, thereby binding more easily to the streptavidin sites in the fluosphere, thus reducing the DNA end labeling efficiency.

A 100,000 MWCO centrifugal filter (10 ml, Amicon Ultra4, Millipore) was used for this purpose. 1X TE buffer was used in this process. First, 1 ml 1X TE buffer is added to the filter. Then, another 1ml 1X TE buffer with 1 μl BSA (10 mg/ml, NEB) was added and the column is centrifuged at 2750g for 4 minutes at 25^oC (Z 300K, Hermle). The filter is taken out, replaced

with 1X TE buffer up to the 2 ml mark of the filter and the centrifugation process was carried out for two more times with the above mentioned conditions.

After this step, the filter was filled with 1ml of 1X TE buffer; ligation solution was added to it slowly using a pipette with wide opened tip. The filter is then filled to the 2 ml mark and centrifuged at 1000g for 16 minutes at 25°C. Such slow speeds are used to avoid shearing of longer DNA molecules. The solution that got drained to the bottom of the filter was removed and the filter was filled with fresh 1X TE buffer to the 2 ml mark. This process was repeated seven times to ensure maximum removal of unbound oligonucleotides.

The final solution retained at the top of the filter was collected and the DNA concentration was measured using a spectrophotometer (NanoDrop, Thermo Scientific) to ensure that the DNA concentration is adequate for the following steps.

<u>Labeling biotinylated λ-DNA with fluospheres:</u>

After the cleanup process, biotinylated λ-DNA was mixed in 1:3 DNA: fluosphere ratio, in 600 ml total volume containing SB-100 buffer (1X TE, 100 mM NaCl, Tween-20, pH 8.0), at 6 rpm, 4°C for 24 hours. We tried DNA end labeling with different types of fluospheres like 200 nm neutravidin fluospheres (F8774, yellow-green fluorescent, Invitrogen) and 40 nm streptavidin transfluospheres (T10711, Red fluorescent, Invitrogen), with later ones being used in most of our experiments.

<u>Labeling DNA molecules without cohesive (cos) sites:</u>

λ-DNA has 12-bp overhanging sites at both ends (<u>*cos* sites</u>), which makes fluosphere end-labeling process easier. Here we used T4 DNA polymerase/Klenow enzyme assisted end labeling for blunt or truncated DNA molecules, showing that our platform can be extended to DNA molecules without *cos* sites. Here, we will discuss in detail with a simple demonstration that adds support to this claim.

For this, we used a combination of T4 DNA polymerase (with 3'-end to 5'-end exonuclease activity, New England Biolabs) and Klenow enzyme (3'-5' exonuclease deleted, New England Biolabs) to label phage T7 DNA, which, unlike lambda DNA, has blunt ends. Analyzing T7 DNA sequence, we found that an adenosine is present only as the 19th base from the 3' end. So, we added a very high concentration of dATP during the T4 DNA polymerase

digestion, so that the digestion process cannot proceed beyond this point. Once the digestion is completed, we added Klenow enzyme with 3'-5' exonuclease activity deleted in presence of dNTPs, where dTTP was replaced by biotin-dUTP. After the reaction, free nucleotides were separated from phage T7 DNA molecules using centrifugal filter cleanup method (which we also used for lambda DNA end labeling process, explained in detail earlier in page 8 of this document.)

As a simple demonstration, we used 2.8 μm streptavidin-coated magnetic beads (Invitrogen) and added biotinylated T7 DNA molecules. Results showed successful end modification using T4 DNAP/Klenow enzyme process, thus DNA molecules binding to the magnetic beads (Fig. S4a). As a control experiment, we used 2.8 μm magnetic beads without streptavidin modification. Results showed no DNA binding to beads, proving that DNA binds to beads only through biotin-streptavidin coupling (Fig. S4b).

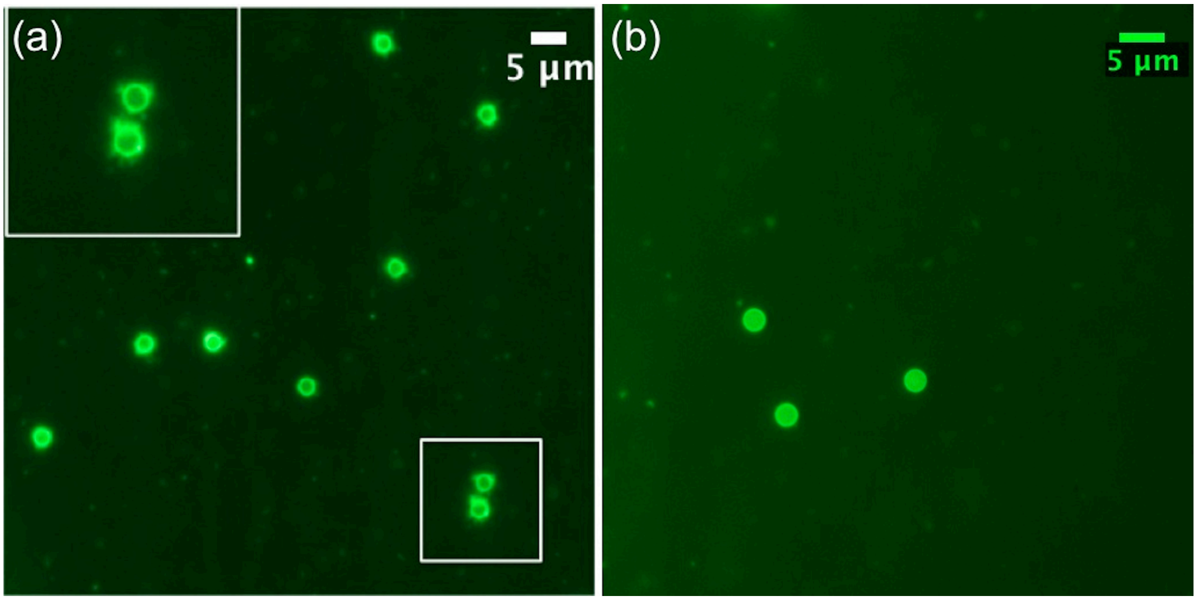

Figure S4. (a) Biotinylated T7 DNA molecules conjugated to 2.8 μm streptavidin-magnetic beads. Boxed region on the lower right is enlarged and shown as an inset at the top left corner. Tens of DNA molecules bind to each bead, as there are more DNA molecules in solution, and each bead has multiple streptavidin binding sites. The scale bar does not apply for the enlarged inset. (b) Control experiment with 2.8 μm magnetic beads without streptavidin modification, showing no binding of T7 DNA molecules.

Next, we also used 40 nm streptavidin transfluospheres to label single DNA molecules. We used a positively charged polylysine coated coverslide to carry out a simple demonstration. Although these experiments were not conducted in our nanoslit devices, it proves that blunt DNA like phage T7 DNA can be labeled using this alternate method (Fig. S5a). Alternately, one could also generate PCR products of any sequence along a DNA strand using biotinylated primers. By using a biotinylated primer in a PCR reaction, the corresponding strand of the PCR product can be biotinylated. To demonstrate end labeling of biotinylated PCR products, we generated a 7 Kb long PCR product using M13 phage DNA as a template, where one end of the PCR product was biotinylated. Later streptavidin-fluospheres and biotinylated 7 Kb PCR products were mixed together for effective end labeling (Fig. S5b).

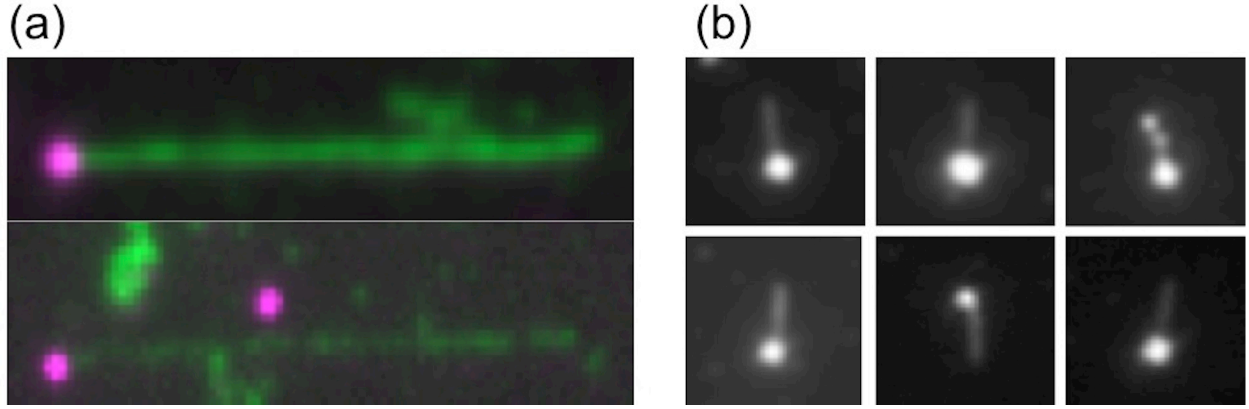

Figure S5. (a) 40 nm fluosphere end labeling of blunt T7 DNA molecules stretched on a polylysine-coated coverslide. (b) 40 nm fluosphere end labeling of biotinylated 7 Kb long PCR products. Biotin was inserted by using a biotinylated primer during the PCR process.

<u>λ-DNA – *E. coli* RNA polymerase holoenzyme (RNAP) complex formation:</u>

Fluosphere labeled λ-DNA (75 pM) was mixed with *E. coli* RNA polymerase holoenzyme (0.5 nM, S90050, Epicentre Biotechnologies) in presence of 5X CLB buffer (0.25 M HEPES-NaOH pH 8.0, 0.5 M NaCl, 25 mM $MgCl_2$, 25% glycerol) in a total volume of 50 µl. All reagents were mixed well using a pipette with wide opened tip to avoid DNA fragmentation. This solution was incubated at 37°C for 15 minutes to form DNA-RNAP open complexes.

Then, 1 µl of 37% formaldehyde (F8775, Sigma Aldrich) was added to the above solution, mixed gently and incubated at 4°C for 30 minutes (4). Formaldehyde reaction was quenched by adding 300 µl of OBS buffer [0.5X TBE (Sigma), 2.5% w/v poly n-vinylpyrrolidone (Sigma),

10% w/v glucose (Sigma) and 0.1% v/v Tween 20 (Sigma)] to this solution. Primary antibody-secondary antibody labeled quantum dot (AB-QD) complex solution (5 nM) prepared separately was added to the above solution of and allowed to mix slowly using rotation (6 rpm) at room temperature in a dark place for an hour.

Formaldehyde induces one atom (2 Å) protein-protein and DNA-protein crosslinks between molecules in close contact. Formaldehyde crosslinking reaction depends on a lot of factors like concentration (of DNA, proteins and formaldehyde), temperature and incubation time. High formaldehyde concentration or long time incubation at high temperatures (like 37°C) leads excessive DNA-protein and protein-protein interactions and in some cases, we even observed formation of blob like structures, in which case, the DNA-RNAP-QD complex could not be stretched with electric field.

Reactions at high temperatures (37°C) were also difficult to control as the reaction is much quicker and even a small change in incubation time lead to undesirable results. So, we used 4°C for formaldehyde cross-linking step to alleviate this problem. One other contributing factor is buffers used for reactions, though a wide variety of buffers with varying salt concentrations have been reported for DNA-RNAP open complex formation step and formaldehyde cross-linking steps (4-7). Finding the right combination of all the parameters to obtain a good working protocol is difficult and we think that the reduced efficiency in the final DNA-RNAP-QD complex is partly due to the complexities involved in formaldehyde crosslinking. Other techniques like UV crosslinking to form in vitro DNA-protein complexes also suffer from reduced efficiency and issues related to extensive crosslinking leading to undesirable results.

<u>AB-QD complex solution preparation:</u>

Primary antibody (Mouse monoclonal, WP001 clone, specific to β' sub-unit, Neoclone Biotech) was diluted from its stock concentration to a final concentration of 1 μM using 1X PBS, pH 7.2. Then, it was mixed with 1 μM quantum dots (Qdot 655 goat F(ab')$_2$ anti-mouse IgG conjugate H+L, Q11022MP, Invitrogen) in 1:1 ratio and incubated at room temperature in a dark place for 45 minutes. The obtained AB-QD solution was used to label RNAP molecules after formation of DNA-RNAP complexes.

<u>Observation Solution:</u>

Above prepared DNA-RNAP-AB-QD complex solution was mixed with equal amounts of OBS buffer solution containing glucose oxidase (50 μg/ml, Sigma), catalase (10 μg/ml, Roche), β-mercaptoethanol (0.5% v/v, Sigma), and YOYO-I dye in DMSO (10 μM, 1:5 dye: base-pair ratio) and allowed to sit at room temperature for 10-15 minutes. This is to ensure proper mixing of dye molecules with the DNA molecules thereby ensuring uniform labeling.

Surface Passivation:

Non-specific binding of biomolecules to micro- and nano-channel surfaces happens to be a serious issue. Especially, non-specifically bound QDs in the nanoslit region in our experiments can be a serious problem. More than DNA molecules, proteins tend to stick to easily to channel surfaces. The situation becomes worse as the surface-to-volume ratio increases. Protein adsorption is also dependent on various factors like type of protein, nature of the surface, pH, temperature, ionic strength of the solution etc. Various groups have tried to address this issue by passivating the surface using traditional methods like saturating the surface with bovine serum albumin (BSA) or caseins. Alternatively, people have also shown improved passivation by coating the surface with polylysine grafted PEG (PLL-g-PEG), lipid bilayers etc. (8)

In our experiments, no separate surface passivation steps were carried out. Rather, we rely upon the high concentration of polyvinylpyrrolidone (PVP, 2.5% w/v) present in our observation solution. Earlier works from Reccius et.al, have demonstrated that PVP indeed reduce non-specific binding of DNA molecules to nanochannel surfaces (9). Here, we tried to see whether it also helps to reduce non-specific interaction of proteins to channel surface. Also, our channel surface is very hydrophilic, which helps to reduce the non-specific binding to a greater extent. Earlier experiments have shown that proteins tend to show adsorb more on hydrophobic surfaces than hydrophilic surfaces (10). Also, studies have shown that increase in protein concentration shows increase in adsorption (11).

To demonstrate that our channels are resistant enough to protein adsorption, the channels were flooded with a very high concentration (50 nM) of streptavidin quantum dots, many folds higher than the concentration used during our DNA-protein complex experiments. QDs were driven from microchannels into the nanoslit region using a small electric field. We did not see any serious non-specific adhesion of QDs to nanoslit surfaces during these experiments. In another experiment, 0.5 ng/μl λ-DNA (no end labeling was done to avoid DNA molecules being

trapped at the micro-nano interface) and 10nM goat anti-mouse IgG QDs (same QDs that were used to label *E. coli* RNAP) together were driven by a small electric field into the nanoslits. No notable sticking of either DNA or anti-mouse IgG QDs were seen in this experiment. (See Movie M5 showing high concentration of streptavidin QDs flooded into the nanoslits and Movie M6 showing λ-DNA and anti-mouse IgG QDs driven into nanoslits using a small electric field.)

Fluorescence Microscopy:

Single molecule imaging was carried out using an inverted epi-fluorescence microscope (Leica DMI-4000B) with a 100X oil objective (plan-Apo, 1.4 N.A., Leica). An additional 1.6X magnifier was used to obtain a field of view of 54x54 μm^2. A mercury lamp was used as the fluorescence excitation source and a custom made filer (470/40nm band-pass/585nm dichroic/655/40nm long-pass filter) was used. A split view system (488nm band-pass/585nm dichroic/655nm long-pass filter, Optical Insights) was used to obtain two channel images for YOYO-I labeled DNA molecules (green) and the end labeled transfluospheres and RNAP labeled with QDs (Red). An electron-multiplied charge-coupled device (EMCCD, Ixon897, Andor) was used to acquire images with an equivalent pixel resolution of 100nm.

Image Processing and Analysis:

DNA length measurements were carried out manually using ImageJ (NIH) software. Distance between end labeled fluosphere and quantum dots were carried out using centroid localization method (12). First, the point spread function (PSF) for our optical setup was determined and images were iteratively deconvolved using a custom macro written in ImageJ. The deconvolved images were then cropped appropriately before further analysis. High precision localization of QDs was carried out by deconvolving the collected distribution of photons (or counts) to the PSF of the system. Using this method, we could achieve localization precision around 2.5 nm for a typical quantum dot point spread function. The position co-ordinate values obtained from this localization were used in finding the distance between two quantum dots.

DNA contour length changes due to YOYO-I dye labeling (for 1:5 dye:base-pair ratio, the contour length increases from 16.5 μm to around 22 μm) (13) and DNA stretching in nanoslits (In our experiments, we achieve around 87% DNA stretching) were taken into account

in calculating the final RNAP binding position values. Distance measurement results obtained from the above steps were converted to values in kilobases.

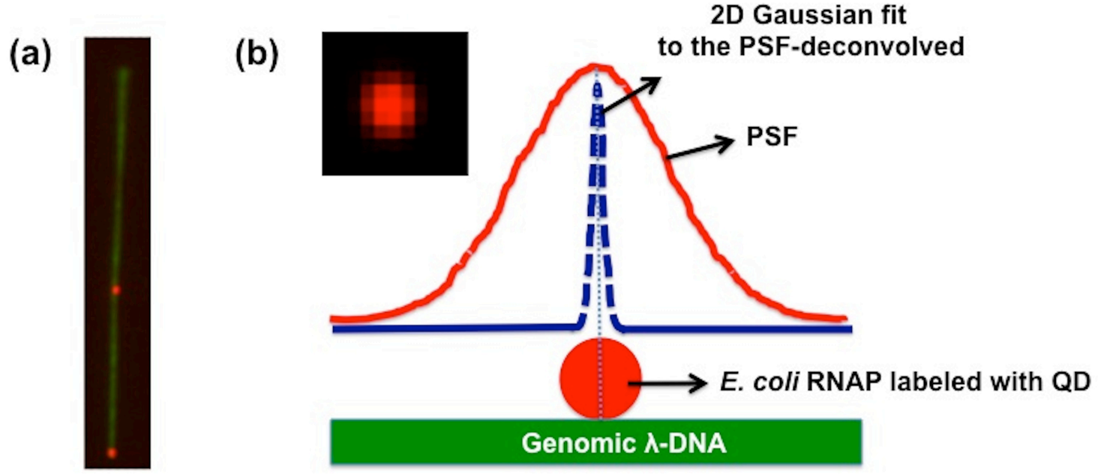

Figure S6. (a) Shows a field-stretched single DNA molecule with QD labeled RNAP used in image analysis. (b) Concept of high resolution QD localization. A quantum dot of around 15 nm size gives a point spread function comparable to its emission wavelength. The centroid co-ordinates can be obtained with greater precision using high resolution localization method. Inset shows a single quantum dot.

Although each slit has 20-30 DNA molecules parallely stretched (See Fig. 2 in main article), only those DNA molecules that has the end labeled fluospheres and at least one RNAP-QD complex bound to stretched DNA were considered for construction of position histograms. Additionally, only DNA molecules 9 μm or longer were considered, such that all expected binding sites are included. This is to avoid any discrepancy in the obtained position histogram.

Results from many molecules (~200) were collected and a histogram was plotted between DNA length (in μm) along X-axis and RNAP binding frequency (counts) along Y-axis using OriginPro 9.0. A multi-peak Gaussian fitting of the obtained histogram was carried out using the PeakAnalyzer function in OriginPro 9.0 (Fig. 4 in main article).

The concept of high precision QD localization is shown in Fig. S6. In this way, we could obtain localization precision around 2.5 nm for a typical QD PSF. The value 2.5 nm refers to the standard error in determining the center or mean value of the distribution. Centroid localization with very high precision has been reported and now a common practice for single molecule experiments. The standard error in determining the center/mean value can be pushed down to 1

nm using Fluorescence Imaging with One Nanometer Accuracy (FIONA) (12,14). We have used the same method to determine the centroid, and the standard error σ is a combination of three terms - photon noise, pixelation and background noise. The equation is as follows:

$$\sigma = [S_i^2/N + [a^2/12]/N + 8\pi S_i^4 b^2/a^2N^2]^{1/2}$$

Among the three terms, the maximum contribution arises from the photon noise term (S_i^2/N), and for the number of photons collected from our experiments (N = 3500), this value is around 2.2 nm. The effective pixel size *a* of the Andor Ixon 897 CCD camera (512 X 512 pixels with size of 16 μm^2/pixel) due to the magnification of 100X oil objective used and the 1.6X additional magnification is 100 nm. So, the pixelation term $[a^2/12]/N$ increases the error to ~2.3 nm. Finally, the third term $8\pi S_i^4 b^2/a^2N^2$, related to the background, accounts for the rest, and a reasonably high signal-to-noise ratio along with background subtraction procedures applied ensured that we have the standard error within 2.5 nm.

Movies:

The supplementary movies are:

Movie M1: Simple animation to show assembly of nanoslit device (fused silica) with PSQ coated coverslide and reservoirs to form a complete working device. This movie corresponds to the last step of supplementary Fig. S1.

Movie M2. Shows fluosphere end labeled λ-DNA molecules trapped at the micro-nano interface and stretched into the nanoslit in the presence of a small DC field. This movie corresponds to Fig. 2 in the main text.

Movies M3. Shows streptavidin-coated QDs moving freely in the nanoslit region without noticeable sticking. This shows effective surface passivation (using PVP in the observation solution) in nanoslits thereby minimizing non-specific binding of proteins on channel surface.

Movie M4. Shows λ-DNA and 655 nm goat anti-mouse IgG QDs moving freely when a small DC field is applied across the nanoslits. This shows that PVP helps to reduce non-specific binding of both DNA and proteins to the channel surface.

Movie M5-M6. Shows YOYO-I labeled λ-DNA-QD labeled *E. coli* RNAP complex stretched in the nanoslit in the presence of a small DC field and recoiled when the field is turned off. These movies correspond to Fig. 4 in the main text.

References

1. Gu, J., Gupta, R., Chou, C.F., Wei, Q. and Zenhausern, F. (2007) A simple polysilsesquioxane sealing of nanofluidic channels below 10 nm at room temperature. *Lab Chip*, **7**, 1198-1201.
2. Finn, R.D., Orlova, E.V., Gowen, B., Buck, M., Heel, M.V. (2000) Escherichia coli RNA polymerase core and holoenzyme structures. *EMBO J.*, **19**, 6833-6844.
3. Perkins, T.T. (1994) Relaxation of a Single DNA Molecule Observed by Optical Microscopy. *Science*, **264**, 822-826.
4. Yu, H. and Schwartz, D.C. (2008) Imaging and analysis of transcription on large, surface-mounted single template DNA molecules. *Anal. Biochem.*, **380**, 111-121.
5. Harada, Y. (1999) Single molecule imaging of RNA polymerase-DNA interactions in real time. *Biophys. J.*, **76**, 709-715.
6. Harada, Y. (2004) Studies on biomolecules using single molecule imaging and manipulation techniques. *Sci. Technol. Adv. Mater.*, **5**, 709-713.
7. Brodolin, K.L., Studitsky, V.M. and Mirzabekov, A.D. (1993) Conformational changes in E.coli RNA polymerase during promoter recognition.pdf. *Nucleic Acids Res.*, **21**, 5748-5753.
8. Persson, F., Fritzsche, J., Mir, K.U., Modesti, M., Westerlund, F. and Tegenfeldt, J.O. (2012) Lipid-based passivation in nanofluidics. *Nano Lett.*, **12**, 2260-2265.
9. Reccius, C.H., Stavis, S.M., Mannion, J.T., Walker, L.P. and Craighead, H.G. (2008) Conformation, length, and speed measurements of electrodynamically stretched DNA in nanochannels. *Biophys. J.*, **95**, 273-286.
10. Nakanishi, K.S., T.; Imamura, K. (2001) On the Adsorption of Proteins on Solid Surfaces, a Common but Very Complicated Phenomenon. *J. Biosci. Bioeng.*, **91**, 233-244.
11. van der Veen, M., Stuart, M.C. and Norde, W. (2007) Spreading of proteins and its effect on adsorption and desorption kinetics. *Colloids Surf. B Biointerfaces*, **54**, 136-142.
12. Qu, X., Wu, D., Mets, L. and Scherer, N.F. (2004) Nanometer-localized multiple single-molecule fluorescence microscopy. *Proc. Natl. Acad. Sci. U.S.A.*, **101**, 11298-11303.
13. Perkins, T.T., Smith, D. E., Larson, R. G., Chu, S. (1995) Stretching of a single tethered polymer in a uniform flow. *Science*, **268**, 83-87.
14. Yildiz, A., Forkey, J.N., McKinney, S.A., Ha, T., Goldman, Y.E. and Selvin, P.R. (2003) Myosin V walks hand-over-hand: single fluorophore imaging with 1.5-nm localization. *Science*, **300**, 2061-2065.
